# Correction to: Social inequality and cultural factors impact the awareness and reaction during the cryptic transmission period of pandemic

**DOI:** 10.1093/pnasnexus/pgaf088

**Published:** 2025-03-18

This is a correction to: Zhuoren Jiang, Xiaozhong Liu, Yangyang Kang, Changlong Sun, Yong-Yeol Ahn, Johan Bollen, Social inequality and cultural factors impact the awareness and reaction during the cryptic transmission period of pandemic, *PNAS Nexus*, Volume 4, Issue 2, February 2025, pgaf043, https://doi.org/10.1093/pnasnexus/pgaf043.

In the originally published version of this article, the basemap for Figure 1b was not derived from an official source. The figure has been updated with a basemap sourced from the National Platform for Common Geospatial Information Service, China, and the figure caption has been updated to reflect this.

