# Supplementary material for: Social inequality and cultural factors impact the awareness and reaction during the cryptic transmission period of pandemic
Source: PNAS Nexus. 2025 Feb 14;4(2):pgaf043. doi: 10.1093/pnasnexus/pgaf043 (PMC11833685; doi:10.1093/pnasnexus/pgaf043)
Supplement: pgaf043_Supplementary_Data [file pgaf043_supplementary_data.pdf]

PNAS nexus

Supplementary Information for

Social inequality and cultural factors impact the awareness and reaction during the cryptic transmission period of pandemic

Zhuoren Jiang, Xiaozhong Liu, Yangyang Kang, Changlong Sun, Yong-Yeol Ahn and Johan Bollen

Corresponding Author: Xiaozhong Liu.
E-mail: xliu14@wpi.edu

This PDF file includes:

Supplementary text
Figs. S1 to S3
Tables S1 to S3
SI References

Supporting Information Text

1. Data Collection

A. Overview. We employ a massive dataset from a major Chinese eCommerce service, Alibaba. Comparing with other kinds of data, like social media data (1, 2), eCommerce dataset have unique advantages to help us intensively and extensively generate implicit/explicate variables. Based on the existing literature (3, 4), *"The initial cases of novel coronavirus (2019-nCoV)–infected pneumonia (NCIP) occurred in Wuhan, Hubei Province, China, in December 2019 and January 2020."* During the cryptic transmission period, without detailed information, COVID-19 can induce awareness/preparedness inequality across China. China also has the one of world's largest eCommerce markets, and the ubiquity of online purchases during the initial outbreak is inescapable (5, 6), which ensures the analysis model's trustworthiness.

The dataset contains information about 800 million individuals and 46.5 billion associated queries, covering 88 days from December 1st, 2019 to February 26th, 2020 (cryptic transmission period). Additionally, we randomly sampled 150 billion historical query/purchase behaviors, shipping addresses, etc. ranging from 2010 to 2019, to estimate individuals' demographics and their social relationships[*]. Furthermore, to explore potential factors such as socio-economic, cultural, structural, resource and epidemic factors that could affect individual behaviors, we collected geolocation (latitude and longitude) information[†] of 366 cities in mainland China, daily confirmed COVID-19 cases information (7), GDP information[‡], cultural tightness[§], the percentage of paddy rice[¶], technology innovation index (10), the proportion of illiterate population aged 15 and above, and the percentage of multi-ethnic households[‖] of 31 provinces in mainland China.

Privacy and Ethical Considerations. While this study leverages extensive e-commerce data to analyze public response and awareness during the early stages of the COVID-19 pandemic, we recognize the ethical concerns associated with using detailed personal data, particularly regarding surveillance and data security.

To address these concerns, we have implemented several robust measures to ensure data privacy and security. Firstly, all data used in this study have been anonymized and de-identified to prevent any possibility of identifying individuals. This includes encrypting any identifiable personal information, such as mobile records, shipping addresses, and GPS data. The data used in this research is stored on a secured server, and access to raw data, including query logs and purchase records, is restricted according to strict data usage agreements. To further ensure privacy, the data cannot be transferred outside of the company's controlled environment, and our team was required to process the data on-site at the company's facilities. Additionally, some sensitive data could not be directly accessed by the authors and was instead anonymized by designated personnel before any analysis was conducted.

Moreover, every step of data querying and analysis underwent rigorous review and approval processes, which included multiple layers of oversight and took significant time to ensure compliance with privacy standards. These measures were designed to protect individual privacy while allowing for meaningful analysis of the data.

Through these extensive privacy protection protocols and transparency in our methods, we aim to responsibly address the ethical concerns related to the use of e-commerce data in this study. By doing so, we contribute to the ongoing discourse on ethical data use in research, particularly in the context of public health emergencies.

IRB. This study was reviewed by the IRB at Indiana University (protocol #: 10521) and was determined not to constitute human subjects research, thus not requiring further review. The experiment and secured data processing methods were also reviewed and approved by Alibaba legal department.

B. The significance of leveraging eCommerce data to analyze early COVID-19 public awareness. The eCommerce data recorded during cryptic transmission period offers a distinctive opportunity to study the evolution of pandemic awareness across various socio-economic groups for several reasons.

- eCommerce has become the primary shopping method for a majority of the Chinese population[**]. This widespread adoption allows for the collection of substantial behavioral data, as opposed to the social interactions and textual expressions typically captured by social media platforms. Such behavioral data provides a more genuine and specific reflection of individual responses and awareness during the early stages of the pandemic, filling the gap left by social media data.
- As a novel data source, eCommerce data allows researchers to explore patterns of pandemic awareness and response from large-scale, real user behaviors. By leveraging advanced technologies such as deep learning, this innovative approach offers robust tools for public health research, particularly in the analysis of vast datasets.
- eCommerce data provides multi-dimensional insights. It encompasses not only purchasing behaviors but also geographic locations, consumption histories, and social recommendations. These diverse data points enable researchers to conduct

[*] All identifiable personal information was removed, and none of the authors had direct access to any individual record. Sensitive information, e.g., shipping and gifting addresses, is encrypted to protect individuals' privacy.

[†] Inquired from https://lbs.amap.com/tools/picker

[‡] Inquired from China Statistical Yearbook 2019, https://www.stats.gov.cn/sj/ndsj/2019/indexeh.htm.

[§] A province-level estimates of cultural tightness from a self-report survey (8).

[¶] The percentage of paddy rice was obtained by calculating the percentage of paddy fields relative to the total cultivated land in each province. These data were sourced from the 1996 China Statistical Yearbook, representing the historical distribution of rice farming and serving as an indicator of social norms (9).

[‖] The proportion of illiterate population aged 15 and above and the percentage of multi-ethnic households are both inquired from China Population Census Yearbook 2020, http://www.stats.gov.cn/tjsj/ndsj/2019/indexeh.htm.

[**] https://www.statista.com/outlook/emo/ecommerce/china

in-depth analyses of different groups' awareness and responses during the pandemic's early stages. Additionally, by examining purchase habits, reviews, and recommendation chains, researchers can infer social network relationships, offering new data sources for understanding the spread of information within social networks.

- eCommerce data collection reflects user behavior in its natural state, free from the influence of research activities. This non-intrusive data collection approach avoids observer effect (11), ensuring the authenticity and reliability of the research outcomes. It is a crucial method for understanding genuine individual behavior.

- eCommerce platforms provide real-time data, capturing a wide array of user purchasing behaviors and search habits instantaneously. This capability allows researchers to monitor public attention to the pandemic and its changing trends dynamically. By analyzing keyword search frequencies and sales data for specific products (such as masks and disinfectants), researchers can track changes in public awareness and capture variations in pandemic perception across different social strata.

- The scale and representativeness of eCommerce data are vast, covering diverse social classes and regions. This broad user base allows for more representative data samples, enhancing the generalizability and reliability of research findings.

- eCommerce data holds potential for early intervention. It can monitor purchasing trends of health-related products before the emergence of new viruses or infectious diseases, providing early signals of public awareness. Early intervention through information dissemination enables public health authorities to take preventive measures more effectively, reducing the potential spread of viruses and improving responses to emerging public health threats.

Overall, using eCommerce data to study individual awareness of COVID-19 during cryptic transmission period in China offers multiple advantages, including data richness, diverse analysis dimensions, methodological innovation, avoidance of observer effects, and potential for early warning. These strengths provide critical support for understanding society's response mechanisms during public health emergencies.

C. Challenges and Timeline in Processing Large-Scale eCommerce Data. We utilized unique and large-scale e-commerce data, making this study the first to employ such data in the context of COVID-19 awareness research. However, this also introduced significant challenges in data acquisition and processing. Firstly, we dealt with an enormous volume of data: from 800 million individuals' 46.5 billion queries, we identified the aware population, ultimately resulting in a dataset of 94 million people for analysis. Additionally, to effectively infer the characteristics of these individuals, we leveraged machine learning techniques to estimate demographic variables and social relationships from 150 billion historical queries and purchase behavior records. This large-scale data processing required considerable time.

Furthermore, we used data from the eCommerce platform Alibaba, which imposed strict data protection and management protocols:

- The data could not leave the company's environment, necessitating that our team send personnel to the company to process the data.

- Due to access restrictions, some sensitive data could not be directly accessed by the authors and had to be queried and anonymized by designated personnel before further processing and analysis, significantly extending the data preprocessing time.

- Every step of data querying and analysis required rigorous reviews and approvals, with each step potentially taking weeks or even months to complete.

2. Estimate Awareness Labels and Individual Features via Data Mining

A. Inferring the awareness label: response to COVID-19. Following prior work (12), the "pandemic awareness" can be considered *"as the possession of information about the outbreak one is willing to act on as opposed to just generally knowing about the disease through media coverage or government programs without taking action."* In this study, we inferred appropriate pandemic awareness labels based on individuals' online shopping behavior for PPE.

During COVID-19 pandemic, the eCommerce purchase and dynamic query logs we collected can honestly reveal individuals' awareness status. For instance, as Table S2 shows, during COVID-19 cryptic transmission period (December 1, 2019 to February 26, 2020), individuals' shopping behavior changed significantly. A prior study (5) has also proven that consumers' fear and anxiety promoted the purchase of PPE during the pandemic.

Given an individual ind_i at time t, we generate the awareness label $L_t^{ind_i}$. If ind_i submitted any query ($q^\star \in \mathbb{Q}$) three or more times at time t, $L_t^{ind_i}$ is set to 1 (aware), otherwise it is set to 0 (not aware). Here, $\mathbb{Q}$ is the collection of COVID-19 related surging queries (high probability during the COVID-19 pandemic and ignorable probability during normal time), specifically those related to personal protective equipment (PPE), i.e., "*(n95/kn95/kf94) & Face mask*".

Once $L_t^{ind_i} = 1$, an individual's awareness status won't change back. For this study, we sampled 94,534,663 individuals (11.8% of all the 800 million individuals we collected) from the Alibaba database for the experiment. All these individuals are individuals with active eCommerce behaviors, and all their awareness labels changed to 1 on or before February 26, 2020, the last day for the experiment.

B. COVID-19 related query collection strategy. Based on World Health Organization (WHO) official documents (13, 14), the initial set of queries that related to COVID-19 disease protection was identified and extracted from the query logs of individuals. As shown in Table S2, the top surging query commodity categories during the COVID-19 outbreak are presented, in search population increment percentage descending order. We, then, choose the most growing category, "*special face masks and respirators*", as $\mathbb{Q}$ for the experiment. Because of the significant growth (search population increment percentage = 102,792% compared to the same period from 2018 to 2019), $\mathbb{Q}$ utilization can minimize data noise for awareness label generation. Furthermore, we only consider the queries that go beyond the necessities of public preparation in the *context of COVID-19*, as advised by the WHO[††]. As shown in Table S1, strict queries like "*(n95|kn95|kf94) & Face mask*" are considered for experiment. Correspondingly, a general query like "*face mask*" would not be utilized for awareness label generation.

C. Characterizing the demographics and social relation features of an individual. By tracking individual ind_i's chronological query/purchase logs, we can generate the demographics and social relation features of an individual with a number of implicit/explicit variables (15) for awareness modeling. For instance, when registering for an Alibaba service, individuals need to provide ID number[‡‡], which explicitly reveals the gender, age, and birth location information. By using their purchase history, we can use machine learning model to estimate individual's implicit feature variables (16, 17), e.g., marital status, child presence, and purchasing power. All the individual variables and the variable descriptions are summarized in Table S3.

Demographic variables. The demographic variables consist of individual-self-reported data and the machine learning model inferred information. For machine learning, massive shopping behavior is used as features for model generation (16). To predict binary variables, such like child presence and marital status, a binary classification model with multi-tasking training (17) is applied for training and prediction. To predict categorical variables, such as occupation, education, and purchasing power, a multi-label classification model with multi-tasking training (17) is applied for training and prediction. The accuracy of machine learning model prediction on the validation dataset is above 95%. For "distance to epicenter" variable, first, the location information of an individual is extracted based on the individual's shipping address[§§], and then the distance to the epicenter is calculated.

Social Networks. By leveraging individual's shipping address, purchase history, etc., three different social networks are generated - Family Network, Schoolmate Network, and Workmate Network, by using machine learning algorithms. For instance, individuals i and j are quite likely to be workmates or schoolmates if they share the same (company or university dorm) shipping address in multiple purchase orders over a period of time. Similarly, when individuals i and j share the same shipping address or regularly order products to each other, they are more likely to be connected in the family network. More detailed network generation algorithms can be found in (18).

Unlike classical social media studies, these networks characterize multi-view and heterogeneous social interactions among different populations/communities, which can be vital to enhance the awareness diffusion models by differentiating various kinds of relations. By using these networks, we can validate relation-type superiority in pandemic awareness diffusion. Furthermore, eCommerce data deduced social networks can be more trustful than other kinds of social media oriented networks, which often carrying weak/noisy connections to pollute the analysis models (19).

D. Statistical Analysis of Awareness Data and Feature Variables. To help readers better understand the awareness data we collected, we analyzed the daily growth rates and cumulative percentages of awareness for all 94,534,663 individuals during the observation period, from December 1st, 2019, to February 26th, 2020 (the cryptic transmission period). Additionally, we calculated the daily growth rates and cumulative percentages for each category of the feature variables of individuals who were already aware. For each day, we identified the highest growth rate and cumulative percentage and listed the corresponding categories of the feature variables. These feature variables include Gender, Age, Occupation, Education, Purchasing Power, Child Presence and Marital Status. The specific definitions of these feature variables can be found in Table S3. For social relations (Family, Schoolmate, and Workmate relations), we calculated the daily growth rates and cumulative percentages of aware social relations and identified the types of social relations with the highest growth rates and cumulative percentages. A social relation is considered aware only if both individuals connected by this relation are aware.

The daily growth rates of awareness for all 94,534,663 individuals, the daily growth rates for each category of feature variables for individuals who were already aware, and the daily growth rates for different aware social relations during the 88-day cryptic transmission period can be accessed at https://doi.org/10.6084/m9.figshare.26831710. The daily cumulative percentages of awareness for all 94,534,663 individuals, the daily cumulative percentages for each category of feature variables for individuals who were already aware, and the daily cumulative percentages for different aware social relations during the 88-day cryptic transmission period can be accessed at https://doi.org/10.6084/m9.figshare.26832400.

Furthermore, we randomly selected a sample of 100,000 individuals from the 94,534,663 to conduct temporal evolutionary logistic regression model analyses. We provided the distributions of all features for individuals who were aware and those who were not aware at 106 critical time points. For feature variables such as Gender, Age, Occupation, Education, Purchasing Power, Child Presence, Marital Status, Family Relation, Schoolmate Relation, and Workmate Relation, we present the percentages of different feature categories at each of the 106 critical time points. For the feature variable "Distance to Epicenter," we report the mean, standard deviation, maximum, and minimum values at each of these time points.

[††] https://www.who.int/emergencies/diseases/novel-coronavirus-2019/advice-for-public
[‡‡] To protect the privacy, all these identifiable personal information is encrypted, and authors cannot access such data.
[§§] The detailed shipping address is encrypted to protect individual privacy.

The distributions of all features for all aware individuals among the 100,000 samples at 106 critical time points can be accessed at https://doi.org/10.6084/m9.figshare.26863462. The distributions of all features for all unaware individuals among the 100,000 samples at 106 critical time points can be accessed at https://doi.org/10.6084/m9.figshare.26863477. The distributions of all features for 100,000 individuals can be accessed at https://doi.org/10.6084/m9.figshare.26863618.

3. Indicator Calculation

A. Percentages of aware people in locations. In this study, we calculate the dynamic population awareness percentages in different locations (i.e., 366 cities or 31 provinces in Mainland China). For a time t, the percentages of aware people in a location l_i can be calculated as:

$$P_{l_i}^{a} = \mathbb{P}_{l_i}^{a} / \mathbb{P}_{l_i}^{total} \tag{1}$$

$P_{l_i}^{a}$ is the percentage of aware people in a location l_i, $\mathbb{P}_{l_i}^{a}$ indicates the aware population in l_i, and $\mathbb{P}_{l_i}^{total}$ indicates the population of individuals who are using the eCommerce platform for a given location l_i.

B. Percentages of aware people in cities. In this study, we calculate the dynamic population awareness percentages in 366 cities of Mainland China. For a time t, the percentages of aware people in a city c_i can be calculated as:

$$P_{c_i}^{a} = \mathbb{P}_{c_i}^{a} / \mathbb{P}_{c_i}^{total} \tag{2}$$

$P_{c_i}^{a}$ is the the percentages of aware people in a city c_i, $\mathbb{P}_{c_i}^{a}$ indicates the aware population in c_i, and $\mathbb{P}_{c_i}^{total}$ indicates the population of individuals who are using the eCommerce platform for a given city c_i.

C. Percentages of aware people with different educational backgrounds. In this study, we calculated the dynamic awareness percentages of people with different educational backgrounds. For a time t, the percentages of aware people in an educational background group e_i can be calculated as:

$$P_{e_i}^{a} = \mathbb{P}_{e_i}^{a} / \mathbb{P}_{e_i}^{total} \tag{3}$$

$P_{e_i}^{a}$ is the the percentages of aware people in an educational background group e_i, $\mathbb{P}_{e_i}^{a}$ indicates the aware population in e_i, and $\mathbb{P}_{e_i}^{total}$ indicates the population of individuals who are using the eCommerce platform for a given educational background group e_i.

D. Social neighborhood awareness ratio (between aware individuals' aware neighbor percentage and unaware individuals' aware neighbor percentage). Following familial, workmate, and schoolmate relations, at time t, for an individual ind, we define P_{ind}^{a,s_i} as the aware neighbor percentage of ind with type s_i social relations:

$$P_{i}^{a,s_i} = \mathbb{P}_{ind}^{a,s_i} / \mathbb{P}_{ind}^{total,s_i} \tag{4}$$

where $\mathbb{P}_{ind}^{a,s_i}$ is the number of aware neighbors of individual ind with s_i social relation, and $\mathbb{P}_{ind}^{total,s_i}$ is the total number of neighbors of individual ind with s_i social relation. For instance, if an individual ind has totally 5 family members, at time t, three of them are already aware of the pandemic, then the awareness percentages of ind with family relation is $P_{ind}^{a,family} = 60\%$.

For a group of aware individuals G_a, $\bar{P}_{G_a}^{a,s_i}$ is the arithmetic mean of all aware individuals' aware neighbor percentages with s_i social-relation:

$$\bar{P}_{G_a}^{a,s_i} = \frac{\sum_{ind_a \in G_a} P_{ind_a}^{a,s_i}}{|G_a|} \tag{5}$$

where $|G_a|$ is the population number of aware individuals in G_a.

Similarly, at time t, for an unaware individual ind_{ua}, we define $P_{i_{ua}}^{a,s_i}$ as the aware neighbor percentage of ind_{ua} with s_i social-relation. Correspondingly, $\bar{P}_{G_{ua}}^{a,s_i}$ is the arithmetic mean of all unaware individuals' aware neighbor percentages within s_i social-relation:

$$\bar{P}_{G_{ua}}^{a,s_i} = \frac{\sum_{ind_{ua} \in G_{ua}} P_{ind_{ua}}^{a,s_i}}{|G_{ua}|} \tag{6}$$

where $|G_{ua}|$ is the population number of aware individuals in G_{ua}.

Then, the neighborhood awareness ratio (between aware individuals' aware neighbor percentage and unaware individuals' aware neighbor percentage) following s_i social-relation, can be calculated as:

$$R_{a/ua}^{s_i} = \frac{\bar{P}_{G_a}^{a,s_i}}{\bar{P}_{G_{ua}}^{a,s_i}} \tag{7}$$

$R_{a/ua}^{s_i}$ can characterize the importance of s_i for pandemic information diffusion, i.e., the larger $R_{a/ua}^{s_i}$ is, the more effective that s_i can contribute to the pandemic information diffusion.

E. Cross-group aware population ratio. In this study, we calculated cross-group aware population ratio R_{po} to measure the ratio between two aware groups:

$$R_{po} = \mathbb{P}_{G_1}^{a} / \mathbb{P}_{G_2}^{a} \tag{8}$$

R_{po} is the cross-group aware population ratio, and $\mathbb{P}_{G_i}^{a}$ indicates the aware population in group G_i.

4. COVID-19 Awareness Data Analysis

A. Gender, marital status & child presence analysis. Figure S1 (a) illustrates the dynamics of awareness regarding the cryptic transmission period of COVID-19, with a specific focus on gender differences. The "zoom in" windows (A and B) provide detailed insights into the growth of awareness among the population during the early period (12/25/2019-01/31/2020) of the COVID-19 pandemic. The trend line for the **Male/Female Ratio** explores chronological changes in the gender awareness ratio, representing the ratio of aware individuals across different gender groups. Notably, the male/female ratio trend did not exhibit a monotonic change, and real-world events were observed to trigger significant ratio changes within a small time window.

For instance, the events "*Wuhan MHC (Wuhan Municipal Health Commission) releasing a briefing about the pneumonia outbreak*" (12/31/2019) and "*Strict exist screening measures activated in Wuhan*" (01/16/2020) led to a decrease in the male/female ratio (from 0.83 to 0.46). Conversely, the event "*The NHC (National Health Commission of China) confirmed human-to-human transmission*" (01/20/2020) resulted in an increase in the ratio. This phenomenon suggests that females may be more sensitive to the early signals of the pandemic, and as its severity escalates (e.g., national-level news releases), males become more actively responsive.

Simultaneously, as the severity of the pandemic reached a certain threshold, the pattern of awareness increase stabilized. For example, the events "*Wuhan lockdown*" (01/23/2020) and "*WHO declared the outbreak of 2019-nCoV a PHEIC*" (01/31/2020) did not alter the significant upward trend of the ratio. These observations align partially with previous epidemiological studies (20, 21), which indicated that females are more susceptible to experiencing panic feelings.

As illustrated in Figure S1 (b), individuals' marital status would affect their awareness patterns. The **Married/Unmarried Ratio** represents the cross-group awareness population ratio. Based on the trends in the married/unmarried ratio, it appears that married individuals may become aware more quickly than unmarried individuals, reaching a peak on 12/30 with a ratio of 1.55. However, as concerns about the epidemic escalated, the growth rate of awareness among unmarried individuals began to accelerate. Eventually, the cumulative number of unmarried aware individuals exceeded the number of married ones after 1/19/2020.

As depicted in Figure S1 (c), the most pronounced distinction lies in the higher likelihood of awareness among individuals with children, which is 10.66 to 5.06 times that of individuals without children, spanning the entire dataset. Moreover, analyzing the trajectory of the **Has Child/No Child Ratio** (cross-group awareness population ratio) suggests that individuals with children tend to become aware more quickly in the early stages, while the awareness responses of individuals without children may be delayed.

B. Occupation, age & purchasing power analysis. The awareness patterns during different phases of a pandemic can be influenced by the population's occupation, purchasing power (income level), and age. As depicted in Figure S2, each sub-graph depicting the awareness pattern on key representative dates across various phases of the pandemic: 12/31/2019 ("*Wuhan MHC released a briefing about the pneumonia outbreak*", Beginning Phase), 01/20/2020 ("*NHC confirmed human-to-human transmission*", Growth Phase), 01/23/2020 ("*Wuhan lockdown*", Peak Phase), and 01/31/2020 ("*WHO declared the novel coronavirus outbreak (2019-nCoV) a Public Health Emergency of International Concern (PHEIC)*", Post-Peak).

For each sub-figure, the upper right corner represents higher purchasing power and a greater awareness percentage, while the lower left area represents lower purchasing power and a lower awareness percentage. The awareness percentage (X-axis) of each occupation group is calculated as follows:

$$P_{o_i}^{a} = \mathbb{P}_{o_i}^{a} / \mathbb{P}_{o_i}^{total} \quad [9]$$

where $P_{o_i}^{a}$ is the awareness percentage of o_i occupation group, $\mathbb{P}_{o_i}^{a}$ is the aware population with occupation o_i, and $\mathbb{P}_{o_i}^{total}$ is the total population with o_i occupation.

Figure S2 can be interpreted as follows: First, during the beginning phase, not surprisingly, hospital staff demonstrated effective consumption of early pandemic signals (0.16% showed awareness), while other groups lagged behind. On average, individuals with high income (high purchasing power) responded more quickly than others. Second, in the growth phase, hospital staff, education/research (including teachers, college students, and researchers), and white-collar company employees exhibited a relatively high awareness percentage (over 2%). However, some low-income groups, such as those in "agriculture, forestry, animal-husbandry, and fishery," and "Individual operation/service staff," displayed a comparatively lower awareness percentage. Third, during the peak phase, the education/research group maintained a relatively high awareness percentage, while individuals from the "agriculture, forestry, animal-husbandry, and fishery" group or blue-collar "worker" group (low purchasing power group) were less aware. Simultaneously, younger individuals demonstrated greater awareness of COVID-19. Similar patterns were also observed in the post-peak phase.

C. Spearman's rank correlation coefficient calculation. In this study, we explore the geographic-related factors that can affect an individual's awareness status, and investigate the factor dynamics. We focus on three main factors: *distance to the epicenter (Wuhan)*, *confirmed COVID-19 case number*, and *local GDP (Gross Domestic Product)*. Additionally, we explore five socio-cultural and structural factors, including: *cultural tightness*, *percentage of paddy rice*, *technology innovation index*, *proportion of illiterate population aged 15 and above*, and *percentage of multi-ethnic households*.

We employ Spearman's rank correlation coefficient (22) to measure the correlation between the ranking of a specific geographic factor and the ranking of geographic awareness percentages[¶¶]. The calculation process is as follows:

[¶¶] Spearman's rank correlation coefficient is computed using the Apache Commons Mathematics Library version 3.6.1 in Java version 1.8.0_31.

- Initially, we generate rankings for geographic locations based on distinct factors.
- Subsequently, we rank the geographic locations according to their awareness percentages for each date.
- For each date, we compute the Spearman's rank correlation coefficient between the ranking of a specific geographic factor and the ranking of geographic awareness percentages.

The awareness percentage of geographic location is calculated by:

$$P_{g_i}^a = \mathbb{P}_{g_i}^a / \mathbb{P}_{g_i}^{total} \quad [10]$$

where $P_{g_i}^a$ is the awareness percentage of geographic location g_i, $\mathbb{P}_{g_i}^a$ is the aware population of g_i, and $\mathbb{P}_{g_i}^{total}$ is the total population of g_i.

For *"distance to the epicenter"*, we use city-level data (totally 366 major cities of Mainland China). For *"confirmed COVID-19 case number"*, *"local GDP"*, *"cultural tightness"*, *"percentage of paddy rice"*, *"technology innovation index"*, *"proportion of illiterate population aged 15 and above"*, and *"percentage of multi-ethnic households"*. we use province-level data (totally 31 provinces of Mainland China). We collected and calculated the geographic awareness percentages of corresponding cities and provinces.

Comprehensive details regarding the daily Spearman's rank correlation coefficients between the ranking of three main geographic factors and geographic awareness percentage ranking and the daily Spearman's rank correlation coefficients between the ranking of five socio-structural, cultural, and resource-related geographic factors and geographic awareness percentage ranking can be accessed at https://doi.org/10.6084/m9.figshare.24131157.

Examining collectivism and awareness. In the main text, we employed cultural characteristics such as cultural tightness and percentage of paddy rice to describe social norms, groups, and interpersonal relationships. These concepts are often associated with collectivism, as collectivist societies typically emphasize adherence to social norms, leading to higher cultural tightness (23, 24). Consequently, we further examined the daily Spearman's rank correlation coefficients between collectivism and awareness across different provinces during the cryptic transmission period. Specifically, we used two provincial-level collectivism indicators: group collectivism and relational collectivism[***]. As shown in Figure S3, we found that both group collectivism and relational collectivism were negatively correlated with awareness. One possible explanation for this result is that it reflects a problem in measuring collectivism through self-report scales (25). Previous studies have found that such methods fail to accurately capture cultural differences (25–27). Comprehensive details regarding the daily Spearman's rank correlation coefficients between the ranking of collectivism geographic factor and geographic awareness percentage ranking can be accessed at https://doi.org/10.6084/m9.figshare.24131157.

5. COVID-19 Awareness Regression Analysis

A. Individual data. In the regression analysis experiment, we conducted random sampling of 100,000 individuals from a pool of 94,534,663 individuals for time-evolving logistic regression models generation. To safeguard privacy, none of the query logs or purchase records in the database used for this study allows for specific identification of individual users, and all individual IDs were encrypted. The query logs and purchase records cannot be shared to ensure privacy protection. To help readers better understand the dynamic changes in awareness among the sampled population throughout the observation period, we share the distributions of all features for all aware individuals among the 100,000 samples at 106 critical time points at https://doi.org/10.6084/m9.figshare.26863462, the distributions of all features for all unaware individuals among the 100,000 samples at 106 critical time points at https://doi.org/10.6084/m9.figshare.26863477, and the distributions of all features for 100,000 individuals at https://doi.org/10.6084/m9.figshare.26863618.

B. Regression model. We generated 106 regression models based on two types of time points:

- The time when the overall awareness percentage increased by 1% (from 1% to 95%).
- The time when 11 impotent real-world events occurred.

The detailed list of time points can be found at https://doi.org/10.6084/m9.figshare.24131085.

For the regression model, the "awareness label $L_t^{ind_i}$" (at time t, individual ind_i's awareness label, 1 for "*aware*" or 0 for "*not aware*") serves as the dependent variable, and ind_i's demographic and social relation features (as summarized in Table S3) serve as the independent variables. We assume a linear relationship between the independent variables (features) and the log-odds of the event that $L_t^{ind_i} = 1$, then establish a logistic regression[†††] (29) as below:

$$\ln\left(\frac{Pr}{1-Pr}\right) = \beta_0 + \beta_1 x_1^{ind_i} + \beta_2 x_2^{ind_i} + \cdots + \beta_n x_n^{ind_i} \quad [11]$$

where β_0 is the intercept, and β_1 represents the variation of $\ln\left(\frac{p}{p-1}\right)$ when x_1 increases by 1, and so on. Detailed information on the 106 regression models can be accessed at https://doi.org/10.6084/m9.figshare.24131109.

[***] A province-level estimates of both group and relational collectivism from a self-report survey (8, 25).

[†††] The logistic regression analysis is performed using statsmodels version 0.11.0 (28) in Python version 3.7.6. The code can be found at https://doi.org/10.6084/m9.figshare.24131130.

6. Face Mask and Pandemic Response

In the context of the ongoing COVID-19 pandemic, understanding public behavior and response mechanisms is crucial for developing effective public health strategies. While numerous studies have explored pandemic response behaviors across different cultural contexts (25, 30–32), such as research (25) noted that by February 4, 2020, 94% of the Chinese population were already wearing masks, these studies primarily focus on the prevalence of preventive behaviors, like mask-wearing, and the cultural factors influencing them. However, they often lack an in-depth analysis of the processes through which public awareness is formed in the early stages of a pandemic.

Our study addresses this gap by analyzing mask-related search behavior on Chinese e-commerce platforms, shedding light on how the public formed awareness through information acquisition and consumption behavior before formal preventive measures were widely implemented. Unlike studies that directly observe mask-wearing behavior, our research emphasizes the dynamics of information dissemination in the early stages of the pandemic, particularly during the cryptic transmission period. This distinct focus not only broadens our understanding of public responses during the pandemic but also highlights how awareness formation in the context of insufficient information dissemination can be influenced by socioeconomic inequalities.

Additionally, cultural factors, particularly cultural tightness and social norms, play a significant role in shaping public awareness and response during pandemics. Previous studies (23, 31–33) have shown that cultural tightness is often associated with higher consistency in social norms and collective action capabilities, which helps increase the adoption rate of public health measures and improve response speed. In our research, we found that regions characterized by higher cultural tightness exhibited significantly greater awareness during the cryptic transmission period of the pandemic, even when the threat was not yet fully understood. This suggests that the influence of cultural tightness extends beyond mere compliance to include enhanced awareness in the face of uncertainty, thereby fostering more effective collective mobilization and social cohesion during critical public health threats. These findings extend the application of cultural dimensions theory and tightness-looseness theory in the context of pandemic awareness, highlighting the complex and dynamic role of cultural factors in shaping public awareness and collective action during times of uncertainty.

Furthermore, while existing research (30) has demonstrated the effectiveness of masks in pandemic prevention, these studies often do not delve into how social inequalities impact awareness formation in the early stages of the pandemic. If social inequalities lead to slower awareness formation among certain groups, this could hinder the timely adoption of effective preventive measures, thereby undermining overall pandemic control efforts. Our study, therefore, not only reveals how socioeconomic inequalities affect early pandemic awareness but also provides a theoretical basis for developing more inclusive and effective public health strategies.

Our research also has unique characteristics. By analyzing data from 94 million individuals and 150 billion search and purchase records on e-commerce platforms, we can capture, in detail, how socioeconomic inequalities affect awareness levels among different groups during the early stages of a pandemic. This research method, based on naturally occurring e-commerce behavior data, avoids self-report bias and observer effects, providing a more accurate reflection of individual decision-making processes. Compared to other studies that primarily rely on survey or aggregate data (25, 30, 31, 34), our approach more precisely reflects actual individual behaviors, revealing the complex social dynamics of the early pandemic period and offering new insights for public health strategy development.

In conclusion, our study, through unique data sources and analytical methods, deeply explores the interaction between information dissemination, cultural factors such as cultural tightness, and socioeconomic inequalities in the early stages of the pandemic. It provides a new perspective on understanding social behavior during the pandemic and offers practical guidance for public health strategy development. Our findings not only broaden the existing literature on pandemic response behaviors but also provide new directions for future research.

Table S1. Comparison of mask-related queries amount and search population

Type	Query Amount	Search Population
All	46,488,164,718	800 millions
Face mask	4,232,403,144 (9.1%)	208,090,096 (26%)
(n95|kn95|kf94) & Face mask	748,105,864 (1.6%)	94,534,663 (11.8%)

Table S2. Increases in search population for coronavirus disease related commodity category query during the COVID-19 pandemic (compared with the same period in previous year).

Query Topic	Increment
Special face masks and respirators	102,792%
Protective suits	17,377%
Disinfectants and medical alcohol	4,678%
Traditional herbal medicines	4,092%
Goggles and Eye protection	2,061%
Thermometer	418%

Table S3. Feature Summary (D: directly parsed from individual reported information; E: estimated information mined by individual's historical behaviors).

Feature	Type	Collection Method	Description
Gender	Categorical	D	Gender of the individual: [Female], [Male]
Age	Categorical	D	Age of the individual: [18-24], [25-29], [30-34], [35-39], [40-44], [45-49], [50-54], [55-59], [$\geqslant$60]
Occupation	Categorical	D & E	Occupation of the individual: [Education/Research], [Hospital staff], [Company employee(white collar)], [Government employee], [Worker(blue collar)], [Individual operation/service staff], [Agriculture, forestry, animal husbandry and fishery]
Education	Categorical	D & E	Individual's highest level of the formal education: [College or Lower], [Bachelor], [Graduate]
Distance to epicenter	Continuous	D	Natural logarithm value of geographical distance (km) from individual's location to epicenter (Wuhan)
Purchasing power	Categorical	E	Purchasing power of the individual: divided into 7 levels from high to low
Child presence	Binary	D & E	Whether there are children present in an individual's household. It identifies the presence of one or more children living within the family unit: 0/1
Marital status	Binary	D & E	If the individual has already married: 0/1
Family relation	Categorical	D & E	At time t, the already aware family members' percentage of the individual: 0:0%,1:(0%-10%],2:(10%-20%],3:(20%-30%],4:(30%-40%],5:(40%-50%],6:(50%-60%],7:(60%-70%],7:(70%-80%],8:(80%-90%],10:(90%-100%]
Schoolmate relation	Categorical	D & E	At time t, the already aware schoolmates' percentage of the individual: 0:0%,1:(0%-10%],2:(10%-20%],3:(20%-30%],4:(30%-40%],5:(40%-50%],6:(50%-60%],7:(60%-70%],7:(70%-80%],8:(80%-90%],10:(90%-100%]
Workmate relation	Categorical	D & E	At time t, the already aware workmates' percentage of the individual: 0:0%,1:(0%-10%],2:(10%-20%],3:(20%-30%],4:(30%-40%],5:(40%-50%],6:(50%-60%],7:(60%-70%],7:(70%-80%],8:(80%-90%],10:(90%-100%]

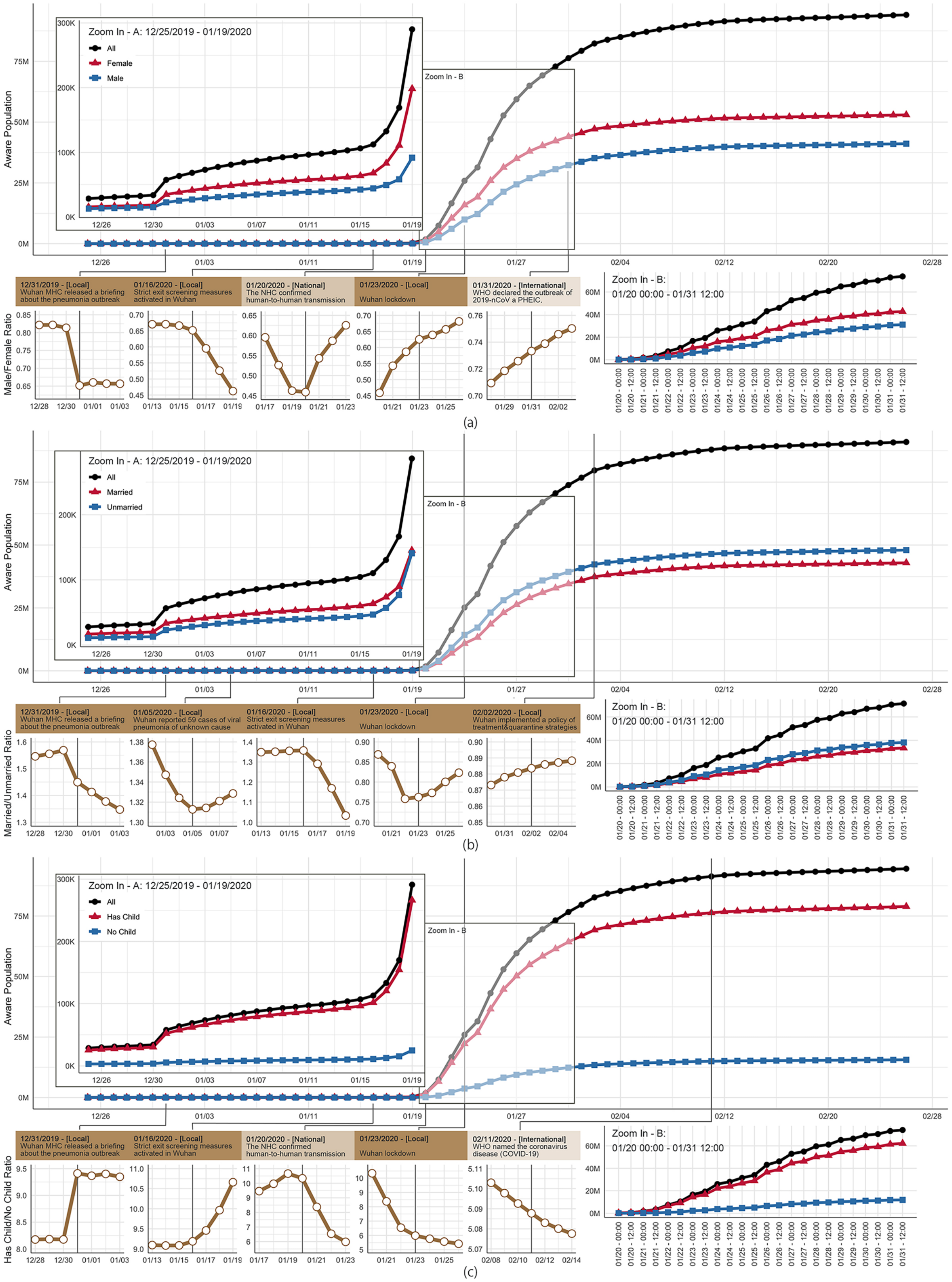

Fig. S1. Time-evolving cross-group aware population ratios, namely (a) Male vs. Female. (b) Married vs. Unmarried and (c) With or without dependents (Child). The "zoom in" windows provide the aware population growth for the an early period (12/25/2019-01/31/2020) of the COVID-19 pandemic. The black curve is the growth curve of the total aware population, the red and blue curves are the growth curves of the aware population across different demographic groups. The bottom left corner of each sub-figure shows the cross-group aware population ratio change triggered by different real-world events in different time windows.

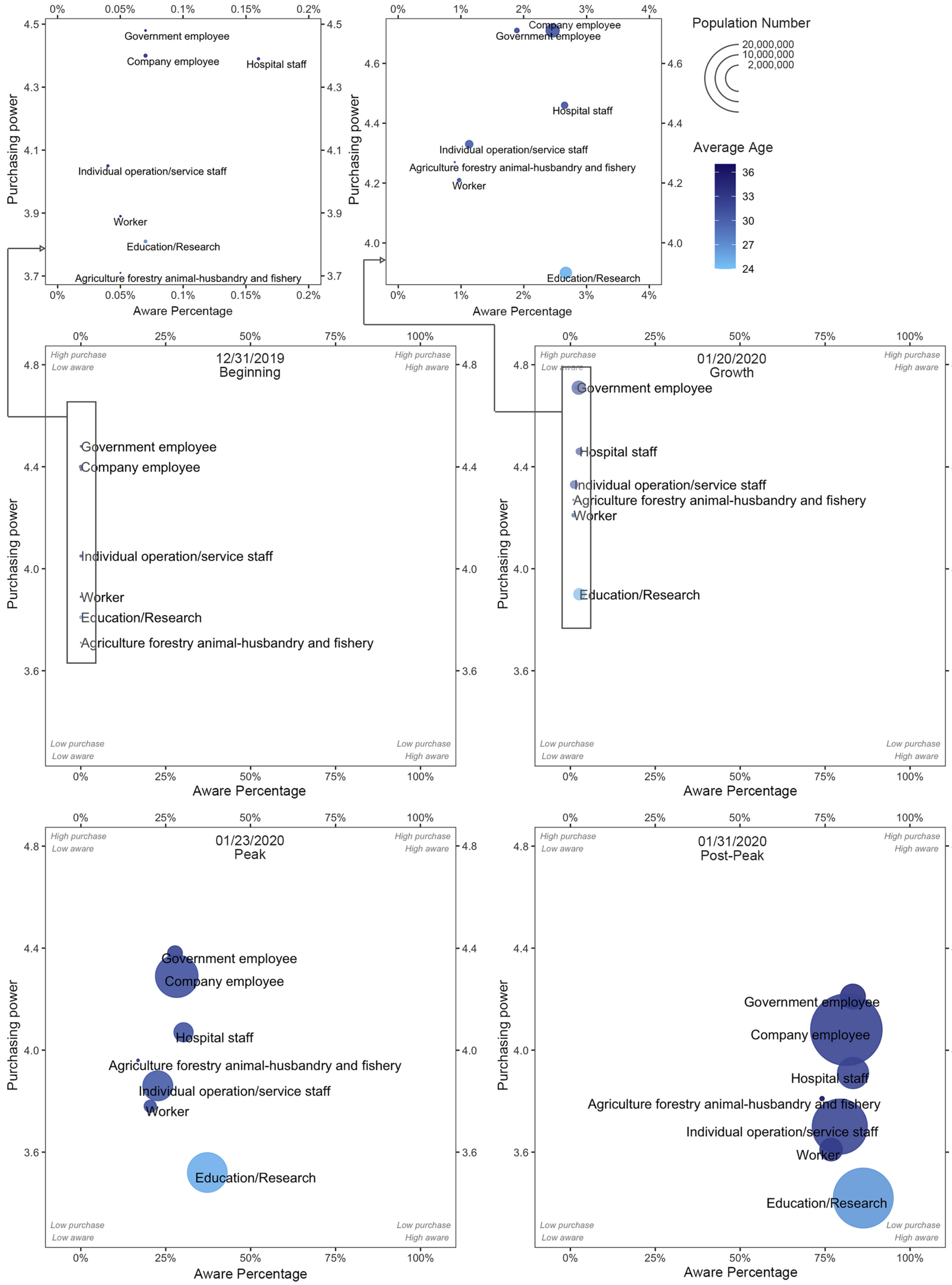

Fig. S2. Awareness Patterns for Different Occupation Groups. Each sub-graph characterizes the awareness pattern of a representative date in a pandemic phase. In each sub-graph, the y-axis represents the purchasing power, the x-axis represents the awareness percentage of different occupation groups. The size of the circle indicates the population of aware occupation group. The color indicates the age distribution within the aware occupation group.

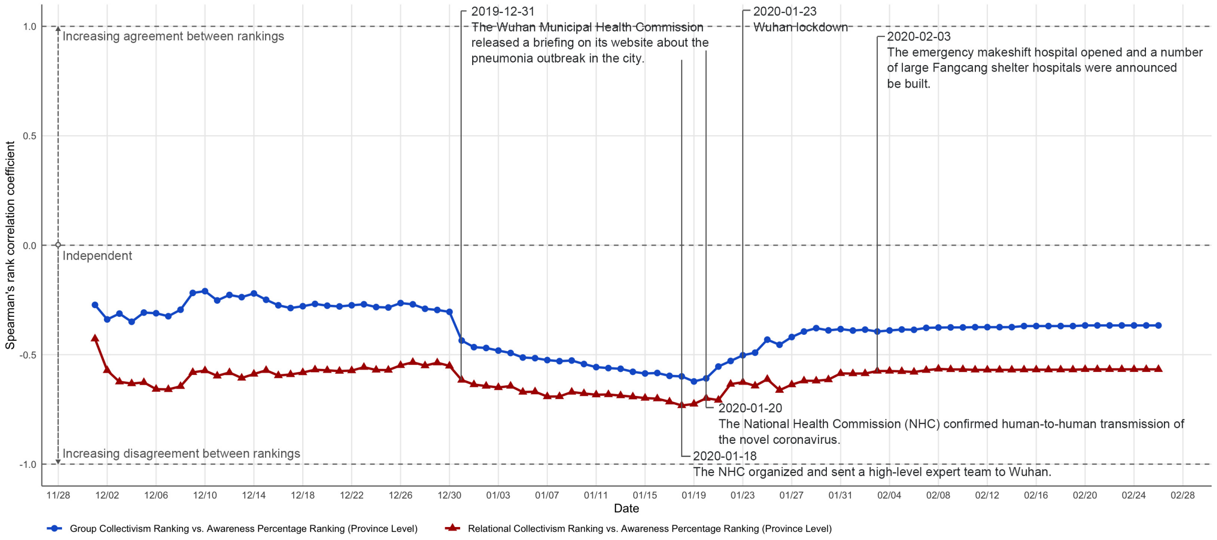

Fig. S3. The Trends of Geography-related Spearman's Rank Correlation Coefficients: Group Collectivism vs. Awareness Percentages (31 provinces) and Relational Collectivism vs. Awareness Percentages (31 provinces).

References

1. DJ Watts, A simple model of global cascades on random networks. *Proc. Natl. Acad. Sci.* **99**, 5766–5771 (2002).
2. SA Myers, C Zhu, J Leskovec, Information diffusion and external influence in networks in *Proceedings of the 18th ACM SIGKDD international conference on Knowledge discovery and data mining.* pp. 33–41 (2012).
3. Q Li, et al., Early transmission dynamics in wuhan, china, of novel coronavirus–infected pneumonia. *New Engl. journal medicine* **382**, 1199–1207 (2020).
4. H Lu, CW Stratton, YW Tang, Outbreak of pneumonia of unknown etiology in wuhan, china: The mystery and the miracle. *J. medical virology* **92**, 401 (2020).
5. PC Addo, F Jiaming, NB Kulbo, L Liangqiang, Covid-19: fear appeal favoring purchase behavior towards personal protective equipment. *The Serv. Ind. J.* **40**, 471–490 (2020).
6. X Gao, X Shi, H Guo, Y Liu, To buy or not buy food online: The impact of the covid-19 epidemic on the adoption of e-commerce in china. *PloS one* **15**, e0237900 (2020).
7. C Huang, et al., Clinical features of patients infected with 2019 novel coronavirus in wuhan, china. *The lancet* **395**, 497–506 (2020).
8. RY Chua, KG Huang, M Jin, Mapping cultural tightness and its links to innovation, urbanization, and happiness across 31 provinces in china. *Proc. Natl. Acad. Sci.* **116**, 6720–6725 (2019).
9. T Talhelm, AS English, Historically rice-farming societies have tighter social norms in china and worldwide. *Proc. Natl. Acad. Sci.* **117**, 19816–19824 (2020).
10. C Science, TDSR Group, C Innovation, UoCAoS Entrepreneurship Management Research Center, *China Regional Innovation Capacity Evaluation Report: 2020.* (Scientific and Technical Document Press), (2020).
11. SK Sporrong, et al., Understanding and addressing the observer effect in observation studies in *Contemporary research methods in pharmacy and health services.* (Elsevier), pp. 261–270 (2022).
12. S Funk, E Gilad, C Watkins, VA Jansen, The spread of awareness and its impact on epidemic outbreaks. *Proc. Natl. Acad. Sci.* **106**, 6872–6877 (2009).
13. WH Organization, , et al., Advice on the use of masks in the context of covid-19: interim guidance, 6 april 2020, (World Health Organization), Technical report (2020).
14. WH Organization, , et al., Rational use of personal protective equipment (ppe) for coronavirus disease (covid-19): interim guidance, 19 march 2020, (World Health Organization), Technical report (2020).
15. KWT Leung, DL Lee, Deriving concept-based user profiles from search engine logs. *IEEE Transactions on knowledge data engineering* **22**, 969–982 (2009).
16. L Guo, et al., Buying or browsing?: Predicting real-time purchasing intent using attention-based deep network with multiple behavior in *Proceedings of the 25th ACM SIGKDD International Conference on Knowledge Discovery & Data Mining.* pp. 1984–1992 (2019).
17. S Xin, et al., Multi-task based sales predictions for online promotions in *Proceedings of the 28th ACM International Conference on Information and Knowledge Management.* pp. 2823–2831 (2019).
18. Y Cen, et al., Trust relationship prediction in alibaba e-commerce platform. *IEEE Transactions on Knowl. Data Eng.* **32**, 1024–1035 (2019).
19. T Baldwin, P Cook, M Lui, A MacKinlay, L Wang, How noisy social media text, how diffrnt social media sources? in *Proceedings of the Sixth International Joint Conference on Natural Language Processing.* pp. 356–364 (2013).
20. B Egloff, SC Schmukle, Gender differences in implicit and explicit anxiety measures. *Pers. Individ. Differ.* **36**, 1807–1815 (2004).
21. S Özdin, Ş Bayrak Özdin, Levels and predictors of anxiety, depression and health anxiety during covid-19 pandemic in turkish society: The importance of gender. *Int. J. Soc. Psychiatry* **66**, 504–511 (2020).
22. W Pirie, Spearman rank correlation coefficient. *Encycl. statistical sciences* **8**, 584–587 (1988).
23. MJ Gelfand, et al., Differences between tight and loose cultures: A 33-nation study. *science* **332**, 1100–1104 (2011).
24. T Talhelm, et al., Large-scale psychological differences within china explained by rice versus wheat agriculture. *Science* **344**, 603–608 (2014).
25. AS English, T Talhelm, R Tong, X Li, Y Su, Historical rice farming explains faster mask use during early days of china's covid-19 outbreak. *Curr. Res. Ecol. Soc. Psychol.* **3**, 100034 (2022).
26. K Peng, RE Nisbett, NY Wong, Validity problems comparing values across cultures and possible solutions. *Psychol. methods* **2**, 329 (1997).
27. SJ Heine, DR Lehman, K Peng, J Greenholtz, What's wrong with cross-cultural comparisons of subjective likert scales?: The reference-group effect. *J. personality social psychology* **82**, 903 (2002).
28. S Seabold, J Perktold, Statsmodels: Econometric and statistical modeling with python in *Proceedings of the 9th Python in Science Conference.* (Austin, TX), Vol. 57, p. 61 (2010).
29. JH McDonald, *Handbook of biological statistics.* (sparky house publishing Baltimore, MD) Vol. 2, (2009).
30. O Damette, TLD Huynh, Face mask is an efficient tool to fight the covid-19 pandemic and some factors increase the probability of its adoption. *Sci. Reports* **13**, 9218 (2023).
31. JG Lu, P Jin, AS English, Collectivism predicts mask use during covid-19. *Proc. Natl. Acad. Sci.* **118**, e2021793118 (2021).
32. S Kitayama, NP Camp, CE Salvador, Culture and the covid-19 pandemic: Multiple mechanisms and policy implications. *Soc. Issues Policy Rev.* **16**, 164–211 (2022).

33. G Hofstede, Culture and organizations. *Int. studies management & organization* **10**, 15–41 (1980).
34. RYN Chung, et al., Socioeconomic inequality in mental well-being associated with covid-19 containment measures in a low-incidence asian globalized city. *Sci. Reports* **11**, 23161 (2021).
